# HIV-Infected Patients: Cross Site-Specific Hydrolysis of H2a and H2b Histones and Myelin Basic Protein with Antibodies against These Three Proteins

**DOI:** 10.3390/biom10111501

**Published:** 2020-10-30

**Authors:** Svetlana V. Baranova, Pavel S. Dmitrienok, Valentina N. Buneva, Georgy A. Nevinsky

**Affiliations:** 1 Institute of Chemical Biology and Fundamental Medicine, Siberian Division of Russian Academy of Sciences, 630090 Lavrentiev, Russia; swb@ngs.ru (S.V.B.); buneva@niboch.nsc.ru (V.N.B.); 2 Pacific Institute of Bioorganic Chemistry, Far East Division, Russian Academy of Sciences, 690022 Vladivostok, Russia; paveldmt@piboc.dvo.ru

**Keywords:** human blood antibodies, HIV infected patients, catalytic antibodies, hydrolysis of H2a, H2b histones, myelin basic protein, cross-complexation and catalytic cross-reactivity

## Abstract

Anti-DNA antibodies are usually produced against histone-DNA complexes appearing during cell apoptosis, while histones are known as damage-associated molecules. A myelin sheath of axons contains myelin basic protein (MBP) playing an important role in the pathogenesis of autoimmune diseases. Antibodies with enzymatic activities (abzymes) are distinctive features of some autoimmune and viral diseases. Abzymes against different proteins can usually only hydrolyze these specific proteins. Using sequential chromatographies of homogeneous IgG preparations from sera of HIV-infected patients on columns with immobilized MBP, H2a, and H2b histones, the anti-MBP, anti-H2a, and anti-H2b antibodies were obtained. It was first shown that IgGs against H2a and H2b effectively hydrolyze these histones and MBP, while anti-MBP split MBP, H2a, and H2b, but no other control proteins. Using the MALDI mass spectrometry, the cleavage sites of H2a, H2b, and MBP by abzymes against these three proteins were found. Among 14 sites of hydrolysis of H2a by IgGs against H2a and 10 sites by anti-MBP IgGs, only one site of hydrolysis was the same for these abzymes. Eleven cleavage sites of H2b with IgGs against H2b and 10 sites of its hydrolysis with antibodies against MBP were different. Anti-H2a, anti-H2b, and anti-MBP abzymes are unpredictable examples of IgGs possessing not only cross-complexation but also catalytic cross-reactivity, which may be a common phenomenon for such abzymes in patients with different autoimmune diseases. The existence of cross-reactivity of abzymes against H2a and H2b histones and MBP represent a great danger to humans since, in contrast with MBP, histones due to cell apoptosis constantly occur in human blood. Anti-H2a, anti-H2b, and anti-MBP can attack and hydrolyze myelin basic protein of the myelin sheath of axons and plays a negative role in the pathogenesis of several pathologies.

## 1. Introduction

It becomes clear during the last three decades that autoantibodies (auto-Abs) from the sera of patients with several autoimmune (AI) as well as several viral diseases similar to artificial abzymes (Abzs) against chemically stable analogs of different chemical reactions (reviewed in [1,2,3]) can possess several different enzymatic activities [3,4,5,6,7,8]. Natural IgGs, IgAs, and IgMs with protease, DNase, RNase, and amylase activities were revealed in sera of patients with several AI and viral diseases [3,4,5,6,7,8,9,10,11,12,13,14,15,16]. Some healthy humans produce Abzs with very low thyroglobulin- [9], VIP- [10], and polysaccharide-hydrolyzing activities [16], but usually healthy humans, as well as patients with many diseases characterizing insignificant autoimmune reactions, lack abzymes [3,4,5,6,7,8]. Germline Abs of healthy humans, however, can exhibit high level superantigen-directed, promiscuous and amyloid-hydrolyzing activities and/or microbe-directed and autoantigen-directed specificities [17,18].

It has been shown that some abzymes could play a significant negative or positive role in forming specific pathogenic patterns in different viral and AI diseases through broadening of their different properties [3,4,5,6,7,8,9,10,11,12,13,14,15]. Abzymes specifically hydrolyze myelin basic protein (MBP), but not other control proteins revealed in the blood of patients with multiple sclerosis (MS) [13,14,15], systemic lupus erythematosus (SLE) [19,20,21], and schizophrenia [22]. These Abzs may play a very negative role in the pathogenesis of these pathologies since they can attack the MBP of the myelin sheath of axons [4,5,6,7,8,13,14,15,19,20,21,22].

Histones play key roles in chromatin functions. However, histones aside from their intranuclear functions are damage-associated molecules, since their administration to animals results in systemic inflammatory and toxic responses, stimulating progression of many diseases, including autoimmune pathologies and cancer [23]. Moreover, anti-DNA antibodies of AI patients are directed mainly against histone-DNA nucleosomal complexes resulting from apoptosis of cells [24]. Apoptotic cells and complexes of DNA with histones are regarded as the primary immunogens and antigens sources in different autoimmune pathologies [24].

Acquired human immune deficiency syndrome (AIDS) is a very dangerous disease ([25] and references therein). The presence of autoimmune phenomena and appearance of autoantibodies in AIDS and some other viral infections could be related to polyclonal B-cell activation, molecular mimicry between viral or microbial and host antigens [25,26,27,28], abnormal expression of immunoregulatory molecules, as well as the anti-idiotypic network [29,30].

Activation of B lymphocytes in HIV-infected patients leads to the production of Abs to viral components and auto-Abs to many different human cell components ([25,30] and references therein). In addition, AIDS patients’ IgGs or IgMs or both, hydrolyze DNA [31], MBP [32], histones [33,34,35,36], HIV-1 integrase [37,38,39,40], and reverse transcriptase [41]. Interestingly, 100% of IgGs of 32 HIV-infected patients efficiently split from one to five human histones (H1, H2a, H2b, H3, and H4) [32,33,34,35,36]. Thus, Abzs against five histones may play a very important negative role in the pathogenesis of AIDS and probably in the pathogenesis of other different AI diseases.

It was recently shown that MBP-hydrolyzing activity is an intrinsic property of IgGs from the blood of HIV-infected patients [32]. It is known from many articles that Abzs against any protein usually only specifically hydrolyze only this, but not many other control proteins ([3,4,5,6,7] and references therein). According to published data, anti-MBP antibodies from sera of patients with several autoimmune diseases can hydrolyze only MBP [3,4,5,6,7,13,14,15,19,20,21,22], while anti-histones Abs can only split histones [33,34,35,36].

Cross-catalytic reactivity between different human proteins can be very dangerous for the development of various autoimmune diseases, including HIV infected patients. It is believed that the development of MS may be associated with people infected with certain bacteria (*Chlamydia pneumonia*, *Staphylococcus*, and *Mycoplasma pneumoniae*) and viruses (human herpesvirus, human endogenous retroviruses, and Epstein–Barr virus) producing different superantigens (for review see [25,26,27,28]). After infection, at first, there can be the production of antibodies against viral and bacterial components, which may be structurally similar to human components [42,43]. Then, due to the mimicry of several viral and bacterial specific proteins with those of human ones, immune system halting can occur, leading to the generation of antibodies against human components resulting in the development of autoimmune diseases.

As yet, there is no literature data on cross-hydrolysis of any proteins by antibodies against other proteins [3,4,5,6,7,8,9,10,11,12,13,14,15]. However, the first exception to this rule has recently been found; it was recently shown that IgGs against H1 histone from sera of HIV-infected patients hydrolyze not only H1 but also MBP and vice versa—abzymes against MBP effectively hydrolyze this histone [32]. However, it could not be excluded that enzymatic cross-activity may not only for be abzymes against MBP and H1 but also against other histones, including H2a and H2b. Histones and their complexes with DNA appear in the blood of healthy and sick people constantly as a result of apoptosis of various cells. When cross-catalytic activity between histones and MBP exists, abzymes against histones can hydrolyze the main myelin protein of axonal envelopes of nerve tissues. This may be an internal factor in the development of disorders of the nervous system in patients with various AI diseases, including HIV-infected patients.

In this study, we revealed enzymatic cross-reactivity IgGs against H2a and H2b histones in the hydrolysis of MBP, while Abs against MBP in the hydrolysis of H2a and H2b histones. Interestingly, IgGs against H2a, H2b, and MBP did not demonstrate catalytic cross-reactivity with respect to other different control proteins. In this article, we have analyzed specific sites of the hydrolysis of H2a and H2b histones by IgGs against these proteins and against MBP using MALDI mass spectrometry.

## 2. Materials and Methods

### 2.1. Chemicals, Donors, and Patients

Protein G-Sepharose and Superdex 200-HR-10/30 columns were from GE Healthcare (GE Healthcare, New York, USA) and homogeneous H2a and H2b histones were provided by Sigma (St. Louis, MO, USA). Human myelin basic protein was purchased from the Molecular Diagnostics and Therapy of DBRC (Moscow, Russia). MBP- and H2a-histone- and H2b-histone-Sepharoses were obtained by immobilization of H2a and H2b histones, and MBP on BrCN-activated Sepharose according to the standard manufacturer’s protocol (St. Louis, MO, USA).

Sera of 29 HIV-infected patients (18–40 y old; men and women) including 13 at the stage of pre-AIDS and 16 at the stage of generalized lymphoadenopathy according to the Center of Disease Control and Prevention classification were used in this study. The protocols of blood sampling meet the guidelines of the human ethics hospital committee (Ethics committee of Novosibirsk State Medical University, Novosibirsk, Russia, permission number 72-H). This committee approved this study in accordance with the guidelines of the Helsinki ethics committee, including a written agreement from patients to present their blood for scientific purposes.

### 2.2. Antibody Purification

Electrophoretically homogeneous IgGs were derived from sera of AIDS patients by chromatography of blood sera proteins using protein G-Sepharose and then by fast protein liquid chromatography (FPLC; gel filtration) in acidic buffer (pH 2.6) on Superdex 200 HR 10/30 column as in [32,33,34,35,36,37,38]. Western blotting of Abs was performed according to [33,34]. The fractions corresponding to the central parts of the IgG peak after gel filtration were concentrated for further chromatographies and assay of catalytic activities. After each step of purification, protein concentrations were measured using a known standard Bradford assay using calibration with bovine serum albumin. To protect from bacterial contamination, IgG preparations were filtered through a Millex filter (pore size 0.1 μm). After storage at +4 °C for 5–7 days for refolding after treatment with acidic buffer, the antibodies were used for their activities assays as described below.

### 2.3. Affinity Chromatography of IgGs on MBP- and Histone-Sepharose

Removal of anti-histone IgGs from total polyclonal Ab preparations was carried out using histone-Sepharose (immobilized equimolar mixture of five histones: H1, H2a, H2b, H3, and H4) column (7 mL) equilibrated in buffer A (20 mM Tris-HCl, pH 7.5) by analogy with [32]. After Abs loading, the column was washed with buffer A to zero optical density. The IgG fraction having no affinity for histones (eluted from histone-Sepharose at loading), was subjected to re-chromatography on MBP-Sepharose to get anti-MBP IgGs. Adsorbed anti-MBP IgGs were eluted using buffer A containing 1.0 M NaCl and finally by acidic buffer (0.1 M glycine-HCl, pH 2.6).

Anti-MBP IgGs were removed from total IgG preparations by affinity chromatography on the MBP-Sepharose column (5 mL) equilibrated in buffer A. The IgG fraction having no affinity for MBP (eluted from MBP-Sepharose at loading) was subjected to re-chromatography on H2a- or H2b-Sepharoses to get anti-H2a and anti-H2b IgGs, respectively. After column washing with buffer A to zero optical density, adsorbed anti-H2a and anti-H2b IgGs were eluted from the sorbents using buffer A containing 3 M NaCl and then with acidic buffer similar to that for MBP-Sepharose as in [32]. IgG fractions eluted from H2a- or H2b-, and MBP-Sepharoses were dialyzed against buffer A and then were used for enzyme-linked immunosorbent assay (ELISA). In addition, in order to completely remove possible anti-H2a and anti-H2b antibody impurities from the anti-MBP antibody preparations, they were passed twice through the H2a-Sepharose column and then twice through the H2b-Sepharose column (in both cases, fractions eluted from the columns upon application were collected). Anti-H2a and anti-H2b IgGs were passed through anti-MBP-Sepharose twice. All IgG preparations before and after their additional purification on alternative sorbents (anti-MBP on anti-H2a and anti-H2b Sepharose), as well as anti-H2a and anti-H2b on MBP Sepharose (see above) showed exactly the same ELISA data (see below).

The relative activity (RA) of IgGs in the hydrolysis of either MBP, H2a, H2b, or all, histones was estimated as described below.

### 2.4. ELISA of Anti-MBP and Anti-Histones Autoantibodies

Anti-MBP, as well as anti-H2a and anti-H2b histones IgGs concentrations were measured using homogeneous preparations of anti-MBP, anti-H2a, and anti-H2b IgGs according to [32,33,34,35,36]. The conditions that we used in this work correspond to the linear parts of the dependence of the ELISA signal on the concentrations of antigens used (40–55% from the plateau). Sodium carbonate buffer (50 μL, pH 9.6) containing 0.01 mg/mL MBP, H2a, or anti-H2b histones was added to ELISA strips for their incubation overnight at 4 °C. The solutions from the wells were removed, then they were washed three times with TBS buffer (20 mM Tris-HCl, pH 7.5, 0.15 M NaCl, 0.05% Triton, and NaN_3_ and 0.05%) and then twice with TBS without Triton X-100. To block the surfaces of the strips, they were treated for 2 h at 37 °C with TBS containing 0.2% egg albumin and 0.01% NaN_3_. The strips were then washed 9 times with water and then with TBS supplemented with 0.01% NaN_3_. TBS containing anti-MBP, anti-H2a, or anti-H2b IgGs, 0.2% egg albumin, 0.01% NaN_3_, and 0.05% Triton X-100 (100 μL) was added to the strips for 2 h (37 °C). After washing all strips with 100 μL water (9 times).

TBS containing egg albumin and NaN_3_ was added for additional incubation for 2 h at 37 °C and washed 9 times with water. Then they were incubated with TBS (100 μL) containing 1 μg/mL conjugate of anti-human monoclonal IgGs with horseradish peroxidase at 37 °C for 30 min and washed 9 times again with water. After the addition of citric-phosphate buffer (50 μL) containing H_2_O_2_ and 3,3′,5,5′-tetramethylbenzidine, all strips were incubated for 15 min at 22 °C; the reaction was stopped by adding of 50% H_2_SO_4_ (50 μL). The relative concentrations of antibodies against MBP, H2a, and H2b histones in anti-MBP, anti-H2a, and anti-H2b IgG preparations were expressed as a difference in optical density (A_450_; an average of 3 measurements) corresponding to samples analyzed with and without the above IgGs against MBP, H2a, and H2b.

### 2.5. Proteolytic Activity Assay

The reaction mixtures (10–20 μL) containing 20 mM Tris-HCl (pH 7.5), 1.0 mg/mL H2a, H2b histones (~13.9 and ~13.78 kDa, respectively) or MBP (~14–18 kDa), and 0.005–0.2 mg/mL anti-H2a, anti-H2b, or anti-MBP IgGs were incubated for 1–10 h at 37 °C as in [32,33,34,35]. Native lactalbumin, human milk lactoferrin, human milk casein, p66 HIV-1 reverse transcriptase (HIV-1 RT), human lysozyme, and human serum albumin were used as control proteins. All three substrates (H2a, H2b, and MBP) are highly basic proteins and may be very sensitive to trypsin-like proteases. Therefore, for additional correct control we denatured lysozyme (thermal denaturation: lysozyme (1 mg/mL) dissolved in deionized water containing 1 mM DTT; 95 °C for 20 min) which is also highly basic and lacks the tertiary structure.

The reactions were stopped, and the efficiency of the hydrolysis of the proteins was analyzed by sodium dodecyl sulfate-polyacrylamide gel electrophoresis (SDS-PAGE) in 4–18% gradient gel under nonreducing conditions. The products of the hydrolysis were detected by Coomassie Blue or silver staining. The gels were first imaged and then scanning and quantified using Image Quant v5.2 software (Media Cybernetics Inc., Rockville, MD, USA). The relative activity of antibodies was evaluated from the loss of these proteins in their initial non-hydrolyzed forms.

SDS-PAGE analysis of enzymatic cross-reactivity of IgGs against MBP, H2a, and H2b was performed using MBP preparation incubation with anti-MBP, anti-H2a, and H2b IgGs.

### 2.6. Kinetic Analysis

The *K*_M_ and *k*_cat_ values were calculated from the dependencies of *V* versus (H2a) or (H2b) by least-squares non-linear fitting using Microcal Origin v5.0 software (Media Cybernetics Inc., Rockville, MD, USA) and presented as linear transformations using a Lineweaver–Burk plot [44]. The *k*_cat_ values (calculated as V_max,_ µM/min/(IgG), µM) are reported as mean ± standard deviation of three independent experiments for each substrate H2a and H2b in their hydrolysis by IgGs (0.04–0.05 mg/mL) against MBP, H2a, and H2b histones. Errors in the values were within 15–20%.

### 2.7. MALDI-TOF Analysis of Ab-Dependent Proteins Hydrolysis

H2a and H2b histones (1.0 mg/mL) were hydrolyzed by anti-H2a-IgGs, anti-H2b-IgGs, or anti-MBP-IgGs (0.005–0.1 mg/mL) for 0–24 h as described above. The analysis of products of the hydrolysis was carried out by MALDI-TOF spectrometry using the Bruker Reflex III system (Bruker Frankfurt, Germany) equipped with a 337-nm nitrogen laser (VSL-337 ND, Laser Science, Newton, MA, USA), 3 ns pulse duration. Mixtures (1 μL) of solution saturated with sinapinic acid (for analysis of proteins ≥10 kDa) or 2-cyano-3-(4-hydroxyphenyl) acrylic acid (for analysis of small peptides and proteins ≤10 kDa) in 0.1% acetonitrile and trifluoroacetic acid (1:2) with 1 μL of the reaction mixtures after hydrolysis of histones and MBP peptides were used for analysis. The final mixtures were applied on the MALDI plates, air-dried, and then used for the study. MALDI-TOF spectra calibrations were carried out using the oligopeptide and protein standards II and I (Bruker Daltonic, Germany) in the internal and external calibration mode. The analysis of histones and MBP peptides cleavage sites was carried out using Protein Calculator v3.3 (Scripps Research Institute).

### 2.8. Analysis of Sequence Homology

The analysis of homology between peptides and protein sequences was carried out using lalign (http://www.ch.embnet.org/software/LALIGN_form.html). This system was used to analyze possible homology between complete protein sequences of MBP with complete H2a and H2b histones.

### 2.9. Statistical Analysis

The results are given as the average mean ± standard deviation of 3 independent experiments for each sample of peptide, proteins, and IgG preparation.

## 3. Results

### 3.1. Purification of Antibodies

We used IgGs of HIV-infected patients isolated first by affinity chromatography of serum proteins using protein G-Sepharose in conditions removing non-specifically bound proteins [32,33,34,35]. Then IgG preparations were additionally purified by FPLC gel filtration under conditions (buffer pH 2.6) destroying immune complexes as in [33,34]. It was shown recently that 100% of IgGs from the sera of 32 HIV-infected patients efficiently hydrolyze from one to five human histones [33,34,35]. In this study, for analysis of catalytic cross-reactivity of IgGs against H2a and H2b antibodies in the hydrolysis of MBP and anti-MBP antibodies in the cleavage of H2a and H2b histones were used polyclonal electrophoretically homogeneous IgG preparations of HIV-infected patients obtained and characterized as in [32]. The mixture of equimolar amounts of 29 IgG preparations (IgG_mix_) with high activity in the hydrolysis of histones and MBP was used for purification of antibodies against H2a, H2b, and MBP, as described above.

It was shown earlier that the nonspecific antigens usually have 1–2 orders of magnitude lower affinity than the specific ones [17,18,19,20,21]. Abs interacting with different affinity sorbents due to cross-complexation can be eluted by 0.1–0.2 M NaCl [17,18,19,20,21]. Total Abs lacking IgGs against MBP was applied on H2a- or H2b-Sepharoses; IgGs with relatively low affinity to these two histones were eluted from the columns with buffer containing 1 M NaCl. Next, fractions of IgGs with a high affinity for H2a or H2b histones were eluted with acidic buffers, pH 2.6. These fractions were used for the analysis of H2a, H2b, and MBP hydrolysis.

To obtain Abs against MBP, the total IgGs fraction after removal of anti-histone IgGs was applied to the MBP-Sepharose column. Anti-MBP IgGs with relatively low affinity was eluted from the column using 1 M NaCl, while having high affinity with acidic buffer, pH 2.6. The fractions with high affinity for MBP were used for the analysis of catalytic cross-reactivity in the hydrolysis of histones and MBP.

### 3.2. ELISA of Anti-MBP and Anti-Histones Autoantibodies

Anti-MBP, as well as anti-H2a and anti-H2b histones IgGs were measured using homogeneous preparations of anti-MBP, anti-H2a, and anti-H2b Abs according to [32,33,34,35,36]. According to ELISA data, Abs against MBP gave a positive answer (A_450_ units) not only against MBP (0.28 ± 0.02) but also against H2a (0.08 ± 0.009) and H2b (0.1 ± 0.01). IgG preparations against H2a demonstrated the following data against different proteins (A_450_ units): H2a (0.26 ± 0.05), H2b (~0.0), and MBP (0.18 ± 0.04). Antibodies against H2b histones demonstrated (A_450_ units): H2b (0.30 ± 0.03), H2a (~0.0), and MBP (0.08 ± 0.009).

Thus, according to ELISA data, anti-H2a IgG preparations did not contain tangible amounts of antibodies against histone H2b and vice versa. However, anti-MBP IgG preparations showed a positive response against H2a and H2b histones, while anti-H2a and anti-H2b Abs against MBP. For any case, we skipped the anti-MBP IgGs twice using columns containing immobilized H2a and H2b histones, while anti-H2a and anti-H2b IgGs Abs through a column with immobilized MBP. However, the ELISA analysis data in all cases completely coincided with that given above. The data obtained could be interpreted in two different ways. On the one hand, the possibility was not excluded that Abs against MBP could not be cleared from IgGs against H2a and H2b histones and vice versa. However, the nonspecific complexation of some proteins with Abs against other ones at ELISA and affinity chromatographies is a widely distributed phenomenon and is known as polyspecificity or polyreactivity of Abs [45,46,47,48]. Catalysis of the transformation of substrates can occur only after the formation of their complexes with abzymes and enzymes. Interestingly, non-specific cross-complexation has also been described for a large number of very different enzymes [49,50,51,52,53]. However, canonic enzymes usually catalyze only one chemical reaction and catalytic conversion by enzymes of unspecific ligands after complexes formation (catalytic cross-reactivity) is an extremely rare case [49,50,51,52,53]. All described data for abzymes against various proteins also hydrolyze only their specific globular proteins [3,4,5,6,7,8,9,10,11,12,13,14,15,19,20,21,22,32,33,34,35,36,37,38,39,40,41]. Therefore, it could be assumed that the criss-crossed complex formation revealed by ELISA could be a consequence of cross-complexation due to the homology of antigenic determinants sequences of MBP with those of H2a and H2b histones. Thus, the ELISA data could not give an answer about the possibility of the presence in each of the IgG preparations of admixtures of Abs to other antigens or implementation of the phenomenon of polyspecificity or polyreactivity. However, in the case of a presence in anti-MBP Abs preparations of IgGs against H2a or H2b abzymes and versa visa, the hydrolysis of H2a or H2b histones should occur at the same specific sites of the protein cleavage. Detection of various H2a and H2b cleavage sites with Abs against these histones, on the one hand, and IgGs against MBP, on the other, may indicate that they possess not only complexation polyreactivity, but also enzymatic cross-activity. Thus, it was very interesting to analyze a possible enzymatic cross-reactivity between Abs-abzymes against H2a and H2b histones and MBP.

### 3.3. SDS-PAGE Analysis of Catalytic Cross-Reactivity

**Figure 1:**
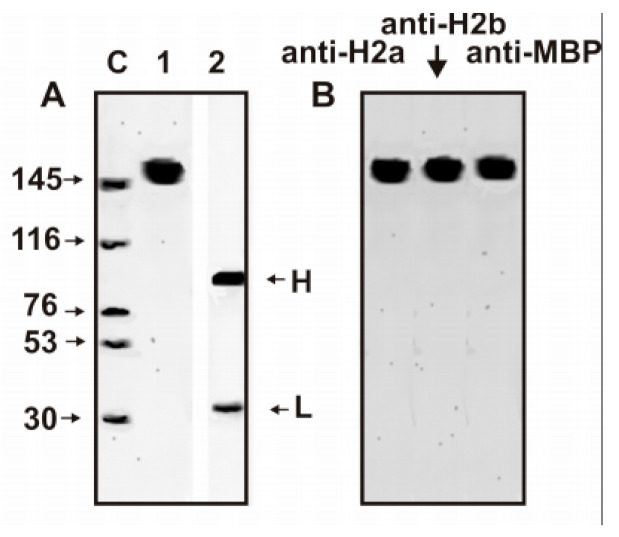
SDS-PAGE analysis of homogeneity of initial IgG_mix_ (10 µg) in 4–18% gradient gel before (lane 1) and after treatment with dithiothreitol (lane 2) (**A**) as well as anti-H2a, anti-H2b, and anti-MBP IgGs after initial IgG_mix_ separation on columns with corresponding affinity sorbents (**B**) followed by silver staining. The arrows (lane C) indicate the positions of molecular mass markers.

**Figure 2:**
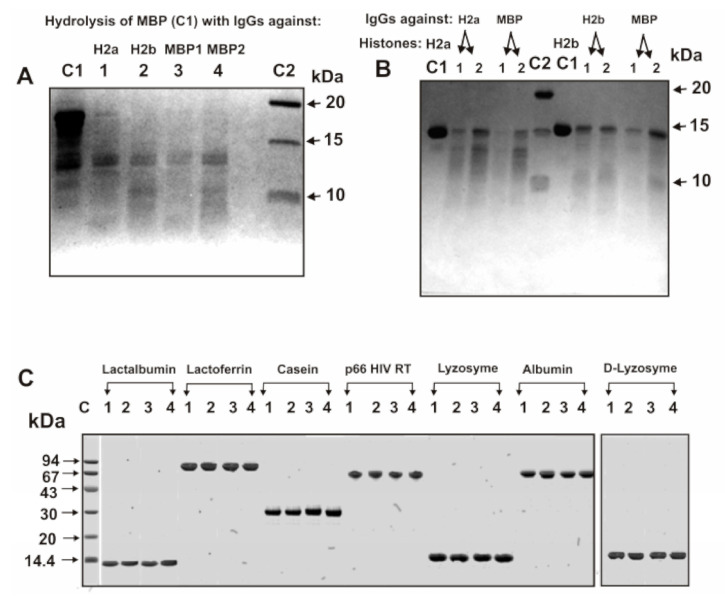
SDS-PAGE analysis of MBP hydrolysis with anti-H2a (lane 1) and anti-H2b (lane 2) IgGs eluted from affinity sorbents with acidic buffer (pH 2.6) as well as splitting of MBP with anti-MBP antibodies eluted from MBP-Sepharose with 0.5 M NaCl (lane 3) and acidic buffer (lane 4) (**A**). SDS-PAGE analysis of H2a hydrolysis with anti-H2a IgGs (lanes 1 and 2) and H2b histone with anti-H2b (lanes 1 and 2) two IgGs preparations eluted from the corresponding affinity sorbents with 0.5 M NaCl and acidic buffer, respectively (**B**). Hydrolysis of H2a and H2b by anti-MBP IgGs eluted from MBP-Sepharose with 0.5 M NaCl and acidic buffer (lanes 1 and 2), respectively, is shown in Panel B. C1 lanes correspond to MBP (**A**), H2a and H2b (**B**) incubated in the absence of IgGs. Lanes C2 correspond to proteins with known molecular masses. The hydrolysis of different proteins in the absence of IgGs (lanes 1), or in the presence of IgGs against H2a (lanes 2), H2b (lanes 3), and MBP (lanes 4) (**C**). Native lactalbumin, human milk lactoferrin, human milk casein, p66 HIV-1 reverse transcriptase (HIV-1 RT); human lysozyme, and human serum albumin and denatured lysozyme (D-Lysozyme) are shown on the Panel C. Lane C corresponds to proteins with known molecular masses.

It was previously shown that even IgG preparations against all five histones (H1–H4), as well as against MBP, hydrolyze only histones and MBP, but not some other control proteins: HIV-1 integrase, HIV-1 reverse transcriptase, bovine serum albumin, human lactoferrin, ferritin, hen egg lysozyme, bovine aldolase, and human milk casein [19,20,21,32,35,39,40,53]. Electrophoretically homogeneous antibodies against H2a, H2b, and MBP obtained in this work (Figure 1) efficiently hydrolyze H2a, H2b, and MBP (Figure 2A,B) but did not hydrolyze six native globular control proteins and denatured lysozyme (Figure 2C).

Unfortunately, electrophoretically homogeneous MBP preparations are not available. Due to alternative splicing of cDNA and partial hydrolysis of protein in the brain of some humans, preparations of MBPs can contain several related protein forms (21.5, 18.5, 17.5, ≤14.0 kDa) and products of their hydrolysis, [54,55]. Line C1 of Figure 2A demonstrates the heterogeneity of the starting preparation of human MBP mainly containing 14–18.5 kDa protein forms. After incubation of this MBP for 24 h with antibodies against H2a, H2b, and MBP, all forms of protein >15 kDa disappear; the formation of smaller peptides is observed (Figure 2A). Noticeable hydrolysis by anti-H2a antibodies of H2b histone and vice versa was not observed during 10 h of the incubation. This correlates with ELISA data on the lack of interaction of anti-H2 antibodies with H2b histone and vice versa. Consequently, one can suppose that not only IgGs against MBP but also against H2a and H2b are able to hydrolyze MBP.

Figure 2B demonstrates that after 24 h the H2a and H2b histones incubation with IgGs against H2a, H2b, and MBP leads to their very efficient hydrolysis. These data are evidence of the possibility of catalytic cross-reactivity of antibodies against MBP, H2a, and H2b. Nevertheless, the H2a and H2b cleavage sites with antibodies against these histones and antibodies against MBP and vice versa may be different. It is important that only the difference in the sites of histone hydrolysis by antibodies against H2a and H2b histones and against MBP can clearly indicate not only their complexation polyreactivity but also the enzymatic cross-activity of antibodies against histones and against MBP.

### 3.4. Analysis of Site-Specificity of H2a Histone Hydrolysis

**Figure 3:**
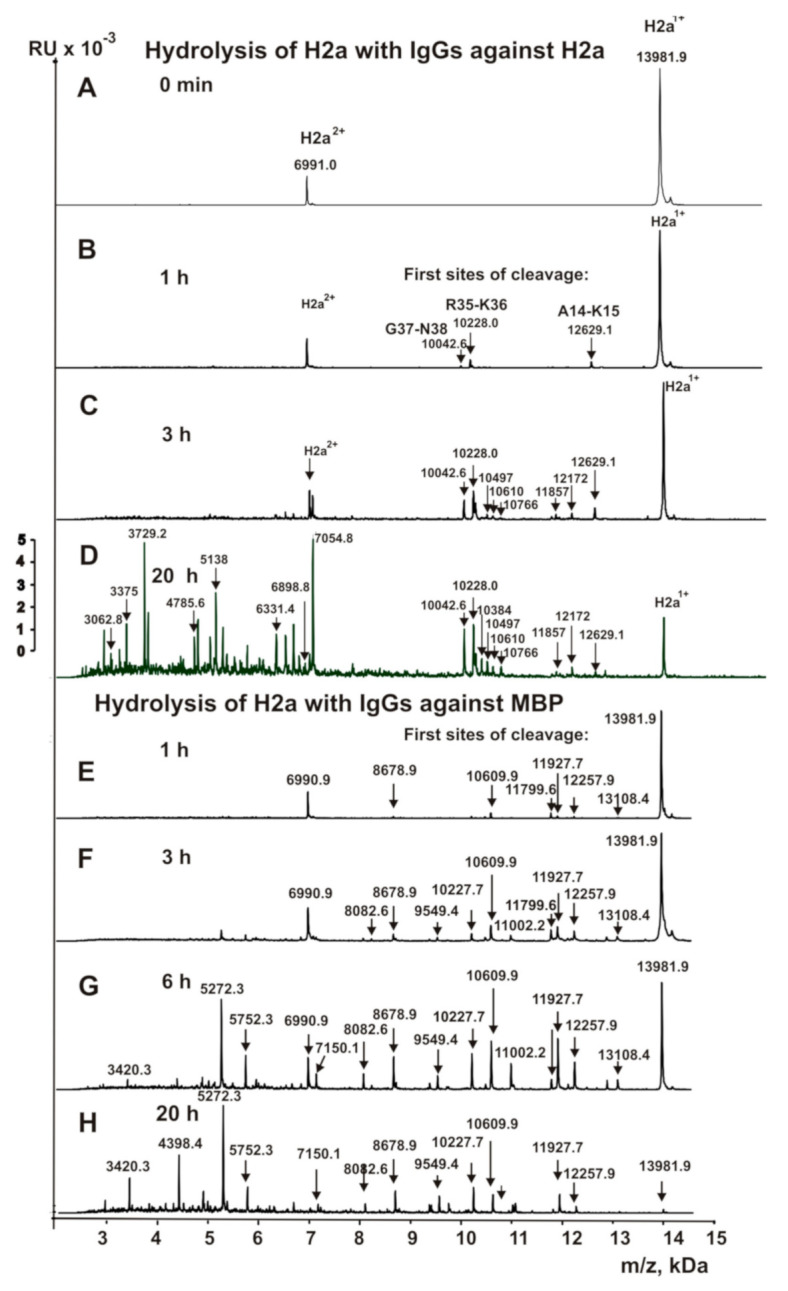
MALDI mass spectra of the H2a (1.0 mg/mL) hydrolysis by IgGs (0.01 mg/mL) against H2a histone (**A**–**D**) and by Abs against MBP (0.02 mg/mL) (**E**–**H**) after different times of the mixtures incubation. Several peaks of short oligopeptides forming after 20 h of the incubation are shown in Panels (**D**,**H**).

To find sites of H2a histone hydrolysis, we analyzed its overtime hydrolysis with IgGs against H2a and MBP eluted from specific sorbents with acidic buffer (pH 2.6). Before addition of IgGs, H2a histone is nearly homogeneous demonstrating its one- (*m*/*z* = 13,981.9 Da) and two-charged ions (*m*/*z* = 6991.0 Da) (Figure 3A). After 1 h of the incubation in the presence of anti-H2a Abs were revealed by only three very small peaks with molecular masses (MMs) of 12,629.1, 10,228.0, and 10,042.6 Da (Figure 3B) corresponding with the following cleavage sites of H2a histone: A14-K15, R35-K36, and G37-N38, respectively (Figure 3B). These three peaks have increased significantly during mixture incubation from 1 to 20 h, and five additional peaks appeared (Figure 3B–D). All eight of these peaks correspond to a cluster of two major (↕) and six moderate (↓) cleavage sites. In addition, there is one cluster of seven very weak cleavage sites (◊) corresponding to the long C-terminal of H2a. After 20 h of the incubation, a lot of peptides with MMs ~2.0–8.0 kDa were revealed (Figure 3D). Most of them are additional products of the cleavage of long oligopeptides (OPs) corresponding to H2a C-terminal part.

**Figure 4:**
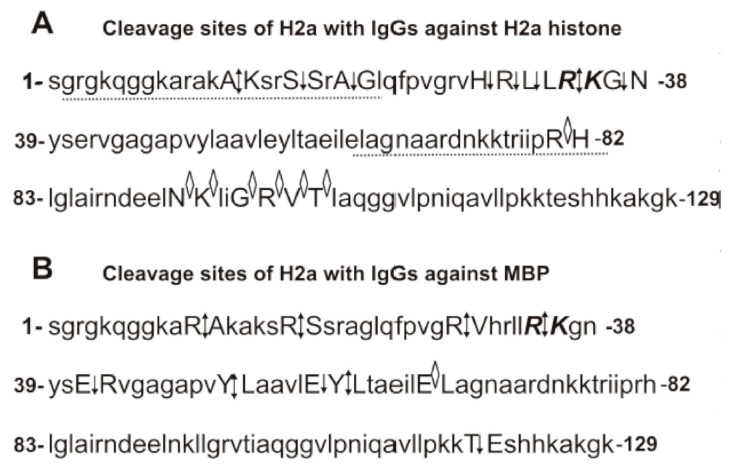
Sites of the H2a hydrolysis by IgGs against H2a histone (**A**) and by Abs against MBP (**B**) revealed using MALDI mass spectra (Figure 3). All major sites of the cleavage are shown by double arrows (↕), moderate ones by simple arrows (↓), and sites of minor hydrolysis by diamonds (◊) (**A**,**B**).

Interestingly, after H2a histone incubation for 1h with IgGs against MBP, six other average or small peaks (Da) were revealed: 8678.9 (Y50–51L), 10,609.9 (Y50-51L), 11,799.6 (R29-V30), 11,927.7 (R20-A21), 12,257.9 (R17-S18), and 13,108.4 (T120-E121) (Figure 3E). The intensity of these peaks grows from 1 to 20 h of the reaction mixture incubation (Figure 3E–G). Moreover, additional peaks were revealed after 3–20 h of the incubation (Da): 8082.6 (E56-Y57), 9549.4 (E56-Y57), 10,227.7 (R35-K36), 11,002.2 (R32-L33) (Figure 3F–H). Interestingly, the four major cleavage sites of H2a with anti-MBP antibodies corresponded to approximately the same protein fragment (from 20 to 38 AAs) as in the case of anti-H2a antibodies (Figure 4A). However, only one site of the hydrolysis (R35-K36) was the same in the case of anti-H2a and anti-MBP abzymes (Figure 4A,B). In addition, in the case of anti-MBP IgGs, there is one cluster of two major, two average, and one weak cleavage sites in the protein zone from 41 to 64 amino acids (AAs), where there are no cleavage sites of H2a by IgGs against this protein. A moderate T120-E121 site of the hydrolysis was also found only in the case of anti-MBP antibodies (Figure 4B). Interestingly, the H2a cleavage sites in the case of antibodies against anti-MBP in the N-terminal zone of the histone do not form a real cluster, and all sites of cleavage correspond to the histone fragment after the R-residue: R-A, R-S, R-V, and R-K (Figure 4B). In the case of six of eight H2a cleavage sites with Abs against this protein correspond to the histone hydrolysis after other amino acids (AAs): A-K, S-S, A-G, H-R, L-L, and G-N (Figure 4A). Hydrolysis of H2a by IgGs against MBP in the central and C-terminal zones of this histone occurs after other AAs: E-R, Y-L, E-Y, Y-L, E-L, and T-E (Figure 4B). A great difference in the sites of H2a hydrolysis with antibodies against this histone and by IgGs against MBP indicates that after binding of histone to these abzymes, they hydrolyze histone showing enzymatic cross-activity.

### 3.5. Analysis of Site-Specificity of H2b Histone Hydrolysis with Abzymes

**Figure 5:**
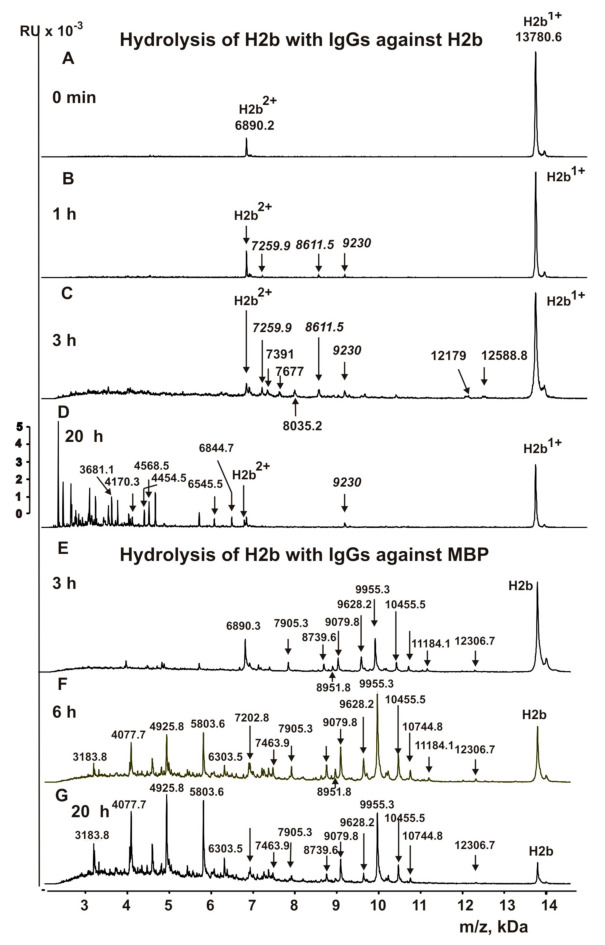
MALDI mass spectra of the H2b (1.0 mg/mL) hydrolysis by IgGs (0.01 mg/mL) against H2a histone (**A**–**D**) and by Abs against MBP (0.02 mg/mL) (**E**–**G**) after different times of the reaction mixture incubation. Several peaks of short oligopeptides forming after 20 h of the incubation are shown in Panels (**D**,**G**).

**Figure 6:**
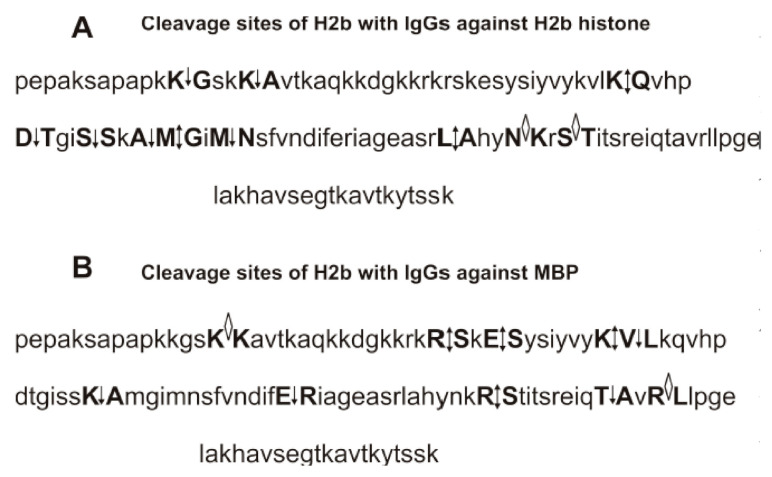
Sites of the H2b hydrolysis by IgGs against H2a histone (**A**) and by Abs against MBP (**B**) revealed using MALDI mass spectra (Figure 5). All major sites of the cleavage are shown by double arrows (↕), moderate ones by simple arrows (↓), and sites of minor hydrolysis by diamonds (◊) (**A**,**B**).

The initial homogeneous H2b histone demonstrates two peaks—the single- (13,780.6 Da) and the double-charged histone (6890.2 Da) (Figure 5A). One hour of incubation leads to the formation of only three small peaks (Figure 5B). After 3 h of the incubation, peptides corresponding to major cleavage sites of H2b by IgGs against this histone have the following MMs (Da): 7259.9 (K46-Q47), 8611.5 (M59-G60), 9230 (L80-A81), 12,578.8 (K12-G13), 12,179 (K16-A17), 8035.2 (D51-T52), 7677.0 (S55-S56), 7391 (A58-M59), and 6958.7 (M62-N63) (Figure 5C). After incubation of the reaction mixture during 20 h, it contains products of a deeper cleavage of long peptides with MMs ~2.0–6.0 kDa (Figure 5D). Four major (R15-S16, E35-S36, K43-V44, and R86-S87), 4 average (V44-L45, K57-A38, E71-R72, and T96-A97), and 2 weak (K15-K16 and R100-L101) sites of H2b hydrolysis by anti-MBP antibodies after 3 h of the incubation were detected (Figure 5E). Figure 5F,G show deeper hydrolysis of H2b hydrolysis during 6 h and 20 h. Among the 11 cleavage sites of H2b with IgGs against H2b (Figure 5E) and 10 sites of its hydrolysis with Abs against MBP, there are none of the same cleavage sites. In addition, in the case of H2b cleavage sites by Abs against this histone, there are extended cluster (P50-H63) sites of the hydrolysis, while for IgGs against MBP there are no other clusters of cleavage sites (Figure 6A,B). Therefore, Abs against H2b and MBP proteins also possess both cross-complexation and enzymatic cross-reactivity.

### 3.6. Analysis of Possible Homology of H2a and H2b Histones with MBP

One of the possible reasons for the catalytic cross-hydrolysis of H2a and H2b histones and MBP by IgGs against these proteins may be a high level of homology of the protein splitting sequences. Supplementary Figure 1 shows the identity of AAs of complete sequences of H2a histone and MBP: 25.9% identity, while similarity of amino acids (non-identical AAs with highly similar physicochemical properties), 51.7%. A nearly similar situation was observed for H2b histone and MBP sequences: 25.7% identity and 53.6% similarity (Supplementary Figure S2).

Interestingly, some of the H2a sequences hydrolyzed by IgGs against H2a and MBP almost match or at least overlap, but the cleavage sites in the fragments of these sequences for anti-H2a and anti-MBP Abs are different (Figure 4). For example, anti-H2a and anti-MBP IgGs hydrolyze the same sequence, but in different sites: anti-H2a IgGs—RAKA↕KSRS↓SRA↓G, while anti-MBP abzymes—R↕AKAKSR↕SSRAG (Figure 4). This H2a histone sequence is homologous to the sequence of MBP (GHHAARTTHYGS): 33% identity and 75% similarity (Figure 4 and Supplementary Figure S1). There is another fragment of H2a sequence (VHRLLRKGN) that is split by both anti-H2a and anti-MBP IgGs but also in different sites. (Figure 4 and Supplementary Figure S1). In addition, there are H2a sequences homologous with those for MBP in which there are cleavage sites only in the case of IgGs against H2a histone or only MBP (Figure 4 and Supplementary Figure S1).

To some extent, a similar situation is observed for the hydrolysis of histone H2b by IgGs against this histone and against MBP (Figure 6 and Supplementary Figure S2). There are fragments of the sequences of H2b and MBP demonstrating increased homology. For example, MBP sequence APKRGSGKDGHHAAR is homologous to APKKGSKKAVTKAQK sequence of H2b: 43.8% identity and 68.8% similarity. This sequence of H2b was hydrolyzed by IgGs against H2b (APKK ↓GSKK↓AVTKAQK) and against MBP (APKKGSK↓KAVTKAQK), but at different specific sites (Figure 6 and Supplementary Figure S2). However, there are only a few good matches between the sites of H2b hydrolysis by antibodies against H2b and MBP (Figure 6, Supplementary Figure S2). Thus, abzymes against H2a and MBP, as well as H2b and MBP, demonstrate enzymatic cross-reactivity. However, cross-hydrolyzable sequences of H2a and H2b by IgGs against these histones and against MBP may overlap or be completely different (Figure 4 and Figure 6, and Supplementary Figures S1 and S2).

### 3.7. Affinity of IgGs for Histones

**Figure 7:**
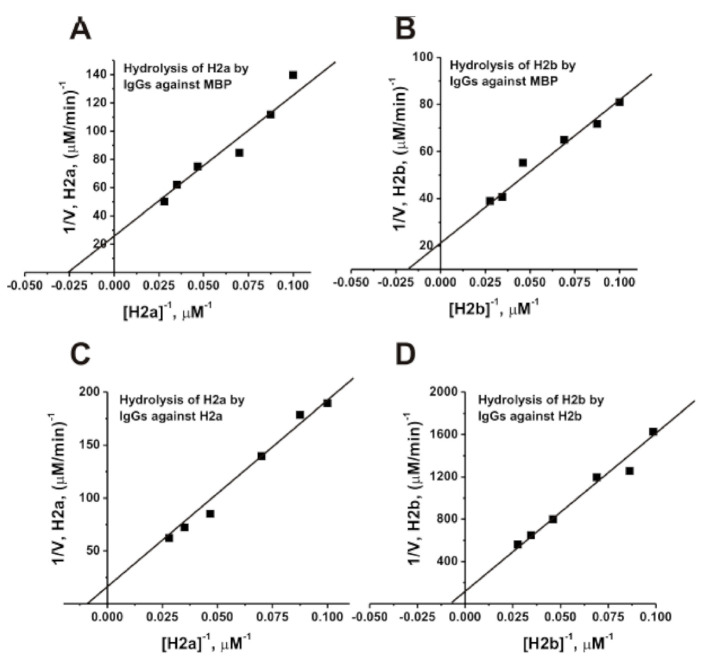
Determination of the *K*_m_ and *V*_max_ (*k*_cat_) values for H2a and H2b substrates in their hydrolysis by IgGs against MBP ((**A**,**B**), respectively; 0.04 mg/mL) and by abzymes against H2a ((**C**); 0.05 mg/mL) and H2b ((**D**); 0.042 mg/mL) using a Lineweaver–Burk plot. The average error in the initial rate determination at each substrate concentration from two independent experiments did not exceed 10–20%.

We have estimated the *K_m_* and *k_cat_* values in the hydrolysis of H2a and H2b by anti-H2a, anti-H2b, and anti-MBP IgGs (Figure 7). The initial rate data obtained at increasing concentrations of H2a and H2b were consistent with the Michaelis–Menten kinetics. The *K*_M_ and *k*_cat_ values for H2a and H2b histones are given in Table 1. The relative affinities of H2a and H2b histones in terms of of *K*_m_ values (41.0 ± 7.0 µM and 59.0 ± 10.0 µM) as well as the relative rates of their hydrolysis by anti-MBP antibodies (0.15 ± 0.025 min^−1^ and 0.18 ± 0.03 min^−1^) are comparable. At the same time, the affinities H2a for anti-H2a IgGs (129.0 ± 19.0 µM) and H2b to anti-H2b IgGs are approximately 2.7–3.2-fold lower than their affinities for IgGs against MBP (Table 1). In addition, the relative rate of H2a hydrolysis (*k_cat_* = 0.33 ± 0.04 min^−1^) by antibodies against this histone is approximately 2.2-fold faster than that for the anti-MBP IgGs (0.15 ± 0.025 min^−1^). It is interesting that IgGs against H2b (0.036 ± 0.006 min^−1^) hydrolyze this histone five times slower than Abs against MBP (0.18 ± 0.03 min^−1^) (Figure 7, Table 1).

## 4. Discussion

Hydrolysis of H2a and H2b by Abs against these histones [33,35] as well as MBP with abzymes against MBP [6,7,8,19,22,32,53] was shown earlier. It was previously shown that pools of anti-MBP Abs contain abzymes of four different types, resembling serine-, thiol-, acidic-like, and metal-dependent proteases, the ratio of which is individual for every autoimmune pathology [4,5,6,7,8,13,14,15,19,20]. A similar situation was observed for abzymes hydrolyzing H2a and H2b histones [33,35].

The nonspecific complexation of some antigens with Abs against other ligands is a widely distributed phenomenon [45,46,47,48,49]. Specific for different substrates, canonic enzymes usually catalyze only one chemical reaction [49,50,51,52,53]. To date, described abzymes against various proteins can hydrolyze only their specific protein [3,4,5,6,7,8,9,10,11,12,13,14,15,19,20,21,22]. In view of this, it was difficult to anticipate that IgGs against any histones and MBP can possess catalytic cross-reactivity. The first example of catalytic cross-reactivity was anti-H1 histone and anti-MBP IgGs from sera of HIV-infected patients [32]. Therefore, in this paper, we analyzed the possible enzymatic cross-reactivity between antibodies-abzymes against H2a and H2b histones and MBP. Using several affinity chromatographies, anti-H2a, anti-H2b, and anti-MBP antibodies were obtained. However, due to the possibility of cross-complexation of IgGs against H2a and H2b histones with MBP (revealed by ELISA), this was not enough to establish enzymatic cross-reactivity. The first evidence of possible enzymatic cross-activity of Abs against two histones and MBP was obtained from the SDS-PAGE analysis of the hydrolysis of H2a, H2b, and MBP with IgGs against these substrates. However, it was obvious that only a significant difference in the hydrolysis sites of the histones by abzymes against H2a, H2b, and MBP can clearly indicate not only their complexation polyreactivity but also the IgGs enzymatic cross-activities.

Fourteen sites of the hydrolysis of H2a by Abs against H2a and 10 splitting sites by IgGs against MBP were revealed (Figure 4). However, only one site of the hydrolysis (R35-K36) was the same in the case of anti-H2a and anti-MBP abzymes (Figure 4). In addition, among the 11 cleavage sites of H2b with IgGs against H2b and 10 sites of its hydrolysis with Abs against MBP, there are no identical cleavage sites (Figure 5).

One of the main reasons for cross-catalysis in the case of abzymes against two histones and MBP can be the homology of their protein sequences. The identity of AAs of complete protein sequences of H2a and H2b histones and MBP is respectively, 25.9% and 25.7% identity, and 51.7% and 53.6% of similarity. Some sites of the hydrolysis of H2a with Abs against this histone and IgGs against MBP to some extent overlap and demonstrate homology with MBP sequences (Supplementary Figure S1). A similar situation was observed for splitting sites of H2a with IgGs against this histone and Abs against MBP (Supplementary Figure S2).

Abzymes against myelin basic protein hydrolyze MBP of axonal envelopes of nerve tissues and therefore are extremely harmful to humans [7]. It is believed that some autoimmune diseases may be a consequence of bacterial and viral infections [32,56,57]. First the immune system can produce antibodies against viral or parasites proteins, and then it may switch to the synthesis of autoantibodies to host antigens due to the molecular mimicry between viral or bacterial and human proteins, alteration of host antigens, abnormal expression of immunoregulatory molecules, and activation of the anti-idiotypic network.

MBP-hydrolyzing abzymes have been detected in the blood of patients with MS [13,14,15], SLE [19,20,21], schizophrenia [22], and HIV-infected patients [32].

MS is a nervous system disease resulting in the manifestation of different psychiatric and nervous disturbances [58]. However, all diseases mentioned above are also more or less related to the violation of the nervous system and mental disorders. Neuropsychiatric disturbances occur in about 50% of patients with SLE [57]. Different violations of synaptic transmission resulting in neuronal damage and serious mental dysfunction in schizophrenia [57] and HIV-infected [58,59] patients were also revealed. Thus, it cannot be excluded that the enzymatic cross-reactivity of Abs hydrolyzing H1 [32].

## 5. Conclusions

We have first shown that IgGs against H2a and H2b histones and against MBP demonstrate not only cross-complexation but also manifest catalytic cross-reactivity. H2a and H2b histones and MBP, which are able to hydrolyze the myelin basic protein of the myelin sheaths of nerve tissues, can probably play an important role in the development of neurodegenerative and neuropsychiatric diseases.

## Figures and Tables

**Table 1:** The *K_m_* and *k_cat_* values for H2a and H2b histones.

Substrate	Abs	*K*_M_, µM	*k*_cat_, min^−1^
H2a	anti-MBP IgGs	41.0 ± 7.0	0.15 ± 0.025
H2b	anti-MBP IgGs	59.0 ± 10.0	0.18 ± 0.03
H2a	anti-H2a IgGs	129.0 ± 19.0	0.33 ± 0.04
H2b	anti-H2b IgGs	160.0 ± 28.0	0.036 ± 0.006

## Supplementary Materials

The following are available online at https://www.mdpi.com/2218-273X/10/11/1501/s1, Figure S1: Analysis of homology between human myelin basic protein and H2a histone, Figure S2: Analysis of homology between human myelin basic protein and H2b histone.

## Abbreviations

AA	amino acid
Ab	antibody
Abz	abzyme
AI	autoimmune
AIDS	acquired immune deficiency syndrome
GL	generalized lymphoadenopathy
AGDs	antigenic determinants
HIV-1	human immunodeficiency virus type 1
OP	oligopeptide
MBP	myelin basic protein
MALDI-TOF	matrix-assisted laser desorption/ionization time-of-flight mass spectrometry
MS	multiple sclerosis
SDS-PAGE	sodium dodecyl sulfate-polyacrylamide gel electrophoresis
SLE	systemic lupus erythematosus

## References

[1] Lerner R.A., Tramontano A. (1987). Antibodies as enzymes. Trends Bioch. Sci..

[2] Schultz P.G., Lerner R.A. (1995). From molecular diversity to catalysis: Lessons from the immune system. Science.

[3] Keinan E. (2005). Catalytic Antibodies.

[4] Nevinsky G.A., Buneva V.N., Keinan E. (2005). Natural catalytic antibodies–abzymes. Catalytic Antibodies.

[5] Nevinsky G.A., Brenner K.J. (2010). Natural catalytic antibodies in norm and in autoimmune diseases. Autoimmune Diseases: Symptoms, Diagnosis and Treatment.

[6] Nevinsky G.A., Kasenga F.H. (2011). Natural catalytic antibodies in norm and in HIV-infected patients. Understanding HIV/AIDS Management and Care—Pandemic Approaches the 21st Century.

[7] Nevinsky G.A., Conzalez-Quevedo A. (2016). Autoimmune processes in multiple sclerosis: Production of harmful catalytic antibodies associated with significant changes in the hematopoietic stem cell differentiation and proliferation. Multiple Sclerosis.

[8] Nevinsky G.A., Khan W.A. (2017). Catalytic antibodies in norm and systemic lupus erythematosus. Lupus.

[9] Kalaga R., Li L., O’Dell J.R., Paul S. (1995). Unexpected presence of polyreactive catalytic antibodies in IgG from unimmunized donors and decreased levels in rheumatoid arthritis. J. Immunol..

[10] Paul S., Volle D.J., Beach C.M., Johnson D.R., Powell M.J., Massey R.J. (1989). Catalytic hydrolysis of vasoactive intestinal peptide by human autoantibody. Science.

[11] Lacroix-Desmazes S., Moreau A., Sooryanarayana, Bonnemain C., Stieltjes N., Pashov A., Sultan Y., Hoebeke J., Kazatchkine M.D., Kaveri S.V. (1999). Catalytic activity of antibodies against factor VIII in patients with hemophilia A. Nat. Med..

[12] Thiagarajan P., Dannenbring R., Matsuura K., Tramontano A., Gololobov G., Paul S. (2000). Monoclonal antibody light chain with prothrombinase activity. Biochemistry.

[13] Polosukhina D.I., Kanyshkova T.G., Doronin B.M., Tyshkevich O.B., Buneva V.N., Boiko A.N., Gusev E.I., Favorova O.O., Nevinsky G.A. (2004). Hydrolysis of myelin basic protein by polyclonal catalytic IgGs from the sera of patients with multiple sclerosis. J. Cell Mol. Med..

[14] Polosukhina D.I., Kanyshkova T.G., Doronin B.M., Tyshkevich O.B., Buneva V.N., Boiko A.N., Gusev E.I., Nevinsky G.A., Favorova O.O. (2006). Metal-dependent hydrolysis of myelin basic protein by IgGs from the sera of patients with multiple sclerosis. Immunol. Lett..

[15] Polosukhina D.I., Buneva V.N., Doronin B.M., Tyshkevich O.B., Boiko A.N., Gusev E.I., Favorova O.O., Nevinsky G.A. (2005). Hydrolysis of myelin basic protein by IgM and IgA antibodies from the sera of patients with multiple sclerosis. Med. Sci. Monit..

[16] Savel’ev A.N., Eneyskaya E.V., Shabalin K.A., Filatov M.V., Neustroev K.N. (1999). Antibodies with amylolytic activity. Protein Pept. Lett..

[17] Paul S., Planque S.A., Nishiyama Y., Hanson C.V., Massey R.J. (2012). Nature and nurture of catalytic antibodies. Adv. Exp. Med. Biol..

[18] Planque S.A., Nishiyama Y., Hara M., Sonoda S., Murphy S.K., Watanabe K., Mitsuda Y., Brown E.L., Massey R.J., Primmer S.R. (2014). Physiological IgM class catalytic antibodies selective for transthyretin amyloid. J. Biol. Chem..

[19] Bezuglova A.V., Konenkova L.P., Doronin B.M., Buneva V.N., Nevinsky G.A. (2011). Affinity and catalytic heterogeneity and metal-dependence of polyclonal myelin basic protein-hydrolyzing IgGs from sera of patients with systemic lupus erythematosus. J. Mol. Recognit..

[20] Bezuglova A.M., Dmitrenok P.S., Konenkova L.P., Buneva V.N., Nevinsky G.A. (2012). Multiple sites of the cleavage of 17- and 19-mer encephalytogenic oligopeptides corresponding to human myelin basic protein (MBP) by specific anti-MBP antibodies from patients with systemic lupus erythematosus. Peptides.

[21] Timofeeva A.M., Dmitrenok P.S., Konenkova L.P., Buneva V.N., Nevinsky G.A. (2013). Multiple sites of the cleavage of 21- and 25-mer encephalytogenic oligopeptides corresponding to human myelin basic protein (MBP) by specific anti-MBP antibodies from patients with systemic systemic lupus erythematosus. PLoS ONE.

[22] Parshukova D., Smirnova L.P., Ermakov E.A., Bokhan N.A., Semke A.V., Ivanova S.A., Buneva V.N., Nevinsky G.A. (2019). Autoimmunity and immune system dysregulation in schizophrenia: IgGs from sera of patients hydrolyze myelin basic protein. J. Mol. Recognit..

[23] Chen R., Kang R., Fan X.-G., Tang D. (2014). Release and activity of histone in diseases. Cell Death Dis..

[24] Fournel S., Muller S. (2002). Antinucleosome antibodies and T-cell response in systemic lupus erythematosus. Ann. Med. Interne.

[25] Fauci A.S., Braunwald E., Kasper D.L., Hauser S.L., Longo D.L., Loscalzo J. (2008). Harrison’s Principles of Internal Medicine.

[26] Ternynck P., Falanga B., Unterkircher C., Gregoire J., Da Silva L.P., Avrameas S. (1991). Induction of high levels of IgG autoantibodies in mice infected with *Plasmodium chabaudi*. Int. Immunol..

[27] Hentati B., Sato M.N., Payelle B., Avrameas S., Ternynck T. (1994). Beneficial effect of polyclonal immunoglobulins from malaria-infected BALB/c mice on the lupus-like syndrome of (NZB x NZW)F1 mice. Eur. J. Immunol..

[28] Matsiota-Bernard P., Mahana W., Avrameas S., Nauciel C. (1993). Specific and natural antibody production during *Salmonella typhimurium* infection in genetically susceptible and resistant mice. Immunology.

[29] Barzilai O., Ram M., Shoenfeld Y. (2007). Viral infection can induce the production of autoantibodies. Curr. Opin. Rheumatol..

[30] Zandman-Goddard G., Shoenfeld Y. (2002). HIV and autoimmunity. Autoimmun. Rev..

[31] Odintsova E.S., Kharitonova M.A., Baranovskii A.G., Siziakina L.P., Buneva V.N., Nevinsky G.A. (2006). DNA-hydrolyzing IgG antibodies from the blood of patients with acquired immune deficiency syndrome. Mol. Biol..

[32] Baranova S.V., Dmitrienok P.S., Buneva V.N., Nevinsky G.A. (2019). Autoantibodies in HIV-infected patients: Cross site-specific hydrolysis of H1 histone and myelin basic protein. Biofactors.

[33] Baranova S.V., Buneva V.N., Nevinsky G.A. (2016). Antibodies from the sera of HIV-infected patients efficiently hydrolyze all human histones. J. Mol. Recognit..

[34] Baranova S.V., Dmitrienok P.S., Ivanisenko N.V., Buneva V.N., Nevinsky G.A. (2017). Antibodies to H1 histone from the sera of HIV-infected patients recognize and catalyze site-specific degradation of this histone. J. Mol. Recognit..

[35] Baranova S.V., Dmitrienok P.S., Ivanisenko N.V., Buneva V.N., Nevinsky G.A. (2017). Antibodies to H2a and H2b histones from the sera of HIV-infected patients catalyze site-specific degradation of these histones. Mol. Biosyst..

[36] Baranova S.V., Dmitrenok P.S., Zubkova A.D., Ivanisenko N.V., Odintsova E.S., Buneva V.N., Nevinsky G.A. (2018). Antibodies against H3 and H4 histones from the sera of HIV-infected patients catalyze site-specific degradation of these histones. J. Mol. Recognit..

[37] Baranova S.V., Buneva V.N., Kharitonova M.A., Sizyakina L.P., Calmels C., Andreola M.L., Parissi V., Nevinsky G.A. (2009). HIV-1 integrase-hydrolyzing antibodies from sera of HIV-infected patients. Biochimie.

[38] Baranova S.V., Buneva V.N., Kharitonova M.A., Sizyakina L.P., Calmels C., Andreola M.L., Parissi V., Nevinsky G.A. (2010). HIV-1 integrase-hydrolyzing IgM antibodies from sera of HIV-infected patients. Int. Immunol..

[39] Odintsova E.S., Baranova S.V., Dmitrenok P.S., Rasskazov V.A., Calmels C., Parissi V., Andreola M.L., Buneva V.N., Zakharova O.D., Nevinsky G.A. (2011). Antibodies to HIV integrase catalyze site-specific degradation of their antigen. Int. Immunol..

[40] Odintsova E.S., Baranova S.V., Dmitrenok P.S., Calmels C., Parissi V., Nevinsky G.A. (2012). Anti-integrase abzymes from the sera of HIV-infected patients specifically hydrolyze integrase but nonspecifically cleave short oligopeptides. J. Mol. Recognit..

[41] Odintsova E.S., Kharitonova M.A., Baranovskii A.G., Siziakina L.P., Buneva V.N., Nevinsky G.A. (2006). Proteolytic activity of IgG antibodies from blood of acquired immunodeficiency syndrome patients. Biochemistry.

[42] Libbey J.E., Cusick M.F., Fujinami R.S. (2014). Role of pathogens in multiple sclerosis. Int. Rev. Immunol..

[43] Andersen O., Lygner P.E., Bergstrom T. (1993). Viral infections trigger multiple sclerosis relapses: A prospective seroepidemiological study. J. Neurol..

[44] Fersht A. (1985). Enzyme Structure and Mechanism.

[45] Zhou Z.H., Tzioufas A.G., Notkins A.L. (2007). Properties and function of polyreactive antibodies and polyreactive antigen-binding B cells. J. Autoimmun..

[46] James L.C., Roversi P., Tawfik D.S. (2003). Antibody multispecificity mediated by conformational diversity. Science.

[47] James L.C., Tawfik D.S. (2003). Conformational diversity and protein evolution—A 60-year-old hypothesis revisited. Trends Biochem. Sci..

[48] James L.C., Tawfik D.S. (2003). The specificity of cross-reactivity: Promiscuous antibody binding involves specific hydrogen bonds rather than nonspecific hydrophobic stickiness. Protein Sci..

[49] Nevinsky G.A. (1995). Important role of weak interactions in long DNA and RNA molecule recognition by enzymes. Mol. Biol..

[50] Nevinsky G.A., Uversky V.N. (2003). Structural, thermodynamic, and kinetic basis of DNA and RNA-dependent enzymes functioning: Important role of weak nonspecific additive interactions between enzymes and long nucleic acids for their recognition and transformation. Protein Structures: Kaleidoscope of Structural Properties and Functions.

[51] Nevinsky G.A. (2011). Structural, thermodynamic, and kinetic basis for the activities of some nucleic acid repair enzymes. J. Mol. Recognit..

[52] Andreev S.L., Buneva V.N., Nevinsky G.A. (2016). How human IgGs against DNA recognize oligonucleotides and DNA. J. Mol. Recognit..

[53] Belov S., Buneva V.N., Nevinsky G.A. (2017). How human IgGs against myelin basic protein (MBP) recognize oligopeptides and MBP. J. Mol. Recognit..

[54] Kamholz J., de Ferra F., Pukkett C., Lazzarini R. (1986). Indentification of three forms of human myelin basic protein by cDNA cloning, *Proc*. Natl. Acad. Sci. USA.

[55] Deibler G.E., Boud L.F., Kies M.W. (1984). Enzymatic and nonemzymatic degradation of myelin basic protein. Neurochem. Res..

[56] O’Connor K.C., Bar-Or A., Hafler D.A. (2001). The neuroimmunology of multiple sclerosis: Possible roles of T and B lymphocytes in immunopathogenesis. J. Clin. Immunol..

[57] Goldner E.M., Hsu L., Waraich P., Somers J.M. (2002). Prevalence and incidence studies of schizophrenic disorders: A systematic review of the literature. Can. J. Psychiatry.

[58] Del Guerra F.B., Fonseca J.L., Figueiredo V.M., Ziff E.B., Konkiewitz E.C. (2013). Human immunodeficiency virus-associated depression: Contributions of immuno-inflammatory, monoaminergic, neurodegenerative, and neurotrophic pathways. J. Neurovirol..

[59] Corcoran C.P. (2003). Neuropsychiatric changes in HIV/hepatitis C coinfected patients undergoing interferon therapy. J. Assoc. Nurses AIDS Care.

