# Supplementary material for: HIV-Infected Patients: Cross Site-Specific Hydrolysis of H2a and H2b Histones and Myelin Basic Protein with Antibodies against These Three Proteins
Source: Biomolecules. 2020 Oct 30;10(11):1501. doi: 10.3390/biom10111501 (PMC7693679; doi:10.3390/biom10111501)
Supplement: Supplementary file 1 [file biomolecules-10-01501-s001.pdf]

Supplementary Figure 1
Analysis of homology between human myelin basic protein and H2a histone

```
Waterman-Eggert score: 131;  11.7 bits; E(1) <  1
25.9% identity (51.7% similar) in 143 aa overlap (46-168:1-113)

           50         60             70          80          90
MBP     SDRGAPKRGSGKDGH-HA- ARTT HY G-S LPQKAQGHRPQDENPVVH- FF     K- NIVTPRTP
        * **  *:* ** :: :*  :*::  : * : :*  : * *       **  ::     *  *  : *:
H2a     SGRG--KQG-GK-ARAKA↕KSRS↓SRA↓GLQFP--V-G-R-------VH↓R↓L↓L↓R↕ KG↓NY-SERV-
                   10           20                          30              40
                   R↓AKAKSR↓S S                     R------↓VH R LL R↓K

     100        110           120        130                140        150
MBP     PPSQGKGRGLSLSR-FSW-G  AEGQKPGFGYGGRASDYKSA----- H-K-GLKGHDAQGTL
            * *  : *:  : :    **  :   :: *: * * *::      * :  ::: :* :  *
H2a     ----GAGAPVYLAA VLE YLTAEILE--LA-GNAARDNKKTRIIPR↓HLQLAIR-NDEE--L
                  50          60            70        80          90
                  Y↓LAAVLE↓YLTAEILE↓L
                  160
MBP     S KIFKLGGR-D-SRS  GS-  P-M-A
        : *:   ** *   :::  *    * : *
H2a     N◊K◊L– LG◊R◊V◊T◊IAQGGVLPNIQA
                  100          110
```

Homology analysis of protein sequences of the myelin basic protein (MBP) and histone H2a was carried out using the program *lalign* (http://www.ch.embnet.org/software/LALIGN_form.html). All identical amino acids of two sequences (H2a and MBP) are marked with an asterisk (*), while non-identical amino acids with highly conserved physicochemical properties are marked with a colon (:). The antigenic determinants sequences of H2a are underlined. The specific sites of H2a histone hydrolysis by antibodies against this histone are indicated in red fragments of H2a sequence: major sites of the cleavage are shown by double arrows (↕), moderate ones by simple arrows (↓), and sites of minor hydrolysis by diamonds (◊). Below the H2a sequence several fragments of this histone and their cleavage sites in the case of anti-MBP antibodies are shown in brown.

Supplementary Figure 2
Analysis of homology between human myelin basic protein and H2b histone

```
>>H2b 125 bp                                                     (125 aa)
 Waterman-Eggert score: 135;  14.0 bits; E(1) <  0.73

25.7% identity (53.6% similar) in 140 aa overlap (50-162:9-125)
      50         60         70         80           90        100
MBP     APKR GSGKDGHH AARTTHYGSLPQKAQGH-RPQD-- ENPVVHFFKNIVTPRTPPPSQ G--
        ***: ** *      *:  *:   :   ** :*: * ::    *:   :: :* ::   :: * :   *
H2b     APKK↓GS-K---K↓AV--TK--A--QKKDGKKRKR SRKESYSIYVYK-VLK↕QVHPD↓T-GIS↓
        10                 20            30         40          50
               SK ◊ K A                          R↕SK E↕S      K↕V↓L
              110                 120        130                     140
MBP     -KGR    GLSL-S----    RFSWGAEGQKPGFG-YGG R A--- S-DYKS AHK---- G-L-KGHD
         *:     *:   *: :     *:: * *:::   :: *:   *:      * : :: * :      * * * *
H2b     SK A↓M↕GIM↓NSFVNDIFE RIA-G-EASR—L↕AHYN◊KR S◊TITSREIQT AVR LLLPGELAK-H-
            60         70            80          90         100
        SK↓AM                FE↓RI                R↕ST         T↓AVR◊L
           150        160
MBP     A--QGTLSK-IFKLGGRDSR
        *   :**   *  :  *     :*:
H2b     AVSEGT--KAVTKY--TSSK
      110          120
```

Homology analysis of protein sequences of the myelin basic protein (MBP) and histone H2b was carried out using the program *lalign* (http://www.ch.embnet.org/software/LALIGN_form.html). All identical amino acids of two sequences (H2b and MBP) are marked with an asterisk (*), while non-identical amino acids with highly conserved physicochemical properties are marked with a colon (:). The antigenic determinants sequences of H2b are underlined. The specific sites of H2a histone hydrolysis by antibodies against this histone are indicated in red fragments of H2a sequence: major sites of the cleavage are shown by double arrows (↕), moderate ones by simple arrows (↓), and sites of minor hydrolysis by diamonds (◊). Below the H2b sequence several fragments of this histone and their cleavage sites in the case of anti-MBP antibodies are shown in brown.
